# Wheel-Running Facilitates Phase Advances in Locomotor and Peripheral Circadian Rhythm in Social Jet Lag Model Mice

**DOI:** 10.3389/fphys.2022.821199

**Published:** 2022-02-16

**Authors:** Satoshi Oneda, Siyuan Cao, Atsushi Haraguchi, Hiroyuki Sasaki, Shigenobu Shibata

**Affiliations:** Laboratory of Physiology and Pharmacology, School of Advanced Science and Engineering, Waseda University, Tokyo, Japan

**Keywords:** circadian clock, social jet lag, behavioral rhythm, peripheral clock, exercise

## Abstract

The circadian clock maintains our health by controlling physiological functions. Social jet lag is one factor that can disrupt the body clock. This is caused by the difference in sleeping hours between weekdays when we live according to social time and holidays when we live according to our body clock. The body clock can be altered by exercise, nutrition, and stress, and several studies have reported that these factors can be used to improve a disturbed body clock. Here we focused on exercise and examined whether continuous wheel-running could improve the disordered body clock in a mouse model that mimics social jet lag. The results showed that the wheel-running exercise group showed faster synchronization of the onset of activities on weekdays which had been delayed by social jet lag and the results were even more pronounced in the high-fat diet feeding condition. Also, when the expression rhythms of the clock genes were examined, they experienced a sudden time shift in the advance light condition or social jet lag condition, it was found that the wheel-running group had a higher ability to adapt to the advance direction. Thus, it is possible that the effective inclusion of exercise in human, especially those who eat high-fat foods, life can improve the disordered body clock in terms of social jet lag.

## Introduction

Circadian clocks are present in almost all organisms and regulate the rhythmicity of their physiological functions that maintain good health (Partch et al., 2014). In particular, the mammalian circadian clock is regulated by a transcription-translation feedback loop of clock genes such as *Cry1/2, Per1/2*, *Bmal1*, *Clock*, and *Rev-erb alpha* (Bass and Takahashi, 2010). In addition, the suprachiasmatic nucleus (SCN) of the hypothalamus is the central clock that regulates body temperature and the sympathetic nervous system of the entire body through a mechanism that appropriately controls the peripheral clocks that exist in the brain and peripheral tissues, excluding the SCN (Moore and Eichler, 1972; Mohawk et al., 2012). Because this central clock is regulated by light signals, light and dark (LD) conditions are important for maintaining the circadian system and homeostasis.

It is important to maintain a healthy lifestyle by following a circadian clock. However, in modern times, changes in light signals from the outside world due to overseas travel, shift work, and changes in activity hours from day to day often create a time lag between the circadian system and the life cycle, which has been shown to potentially disrupt the circadian clock system (Désir et al., 1982; Skene et al., 1996). Moreover, even if an individual does not travel abroad or work shifts frequently, disruption of the circadian clock system can still occur. One of the most common is the so-called social jet lag (SJL). Similar to traveling abroad or shift work, SJL causes time lags and disruptions between the circadian system and the life cycle (Wittmann et al., 2006). The accumulation of SJL is thought to be the cause of its association with various diseases (Roenneberg et al., 2012; Stoner et al., 2018; Henderson et al., 2019; Borgio and Louzada, 2021). Therefore, it is important to identify effective ways to improve the SJL-disrupted body clock to maintain health. A previous paper reported the elimination of jet lag and shift work via chocolate in the morning, but no clear method of eliminating SJL has been reported (Escobar et al., 2020).

Here we focused on exercise in mice to improve the time lag between the circadian clock system and the living environment, including SJL. Exercise, a factor that regulates physiological functions such as body temperature and hormone secretion, is reportedly related to the biological clock (Tahara et al., 2017a). Several studies have shown that exercise can shift the circadian rhythm phase in mice (Reebs and Mrosovsky, 1989; Marchant and Mistlberger, 1996). In addition, wheel-running alters the expression of the clock genes *Per1* and *Per2* in the SCN, while other studies reported that regular exercise under constant dark (DD) conditions can replace light stimulation as a cue to maintain a constant circadian system cycle (Maywood et al., 1999; Hughes et al., 2021). On the other hand, in the peripheral organs, forced exercise resets the body clock, while the wheel-running exercise for a few days in the middle of the light period advances the phase of the clock gene expression rhythm in the liver and kidney by approximately 3 h (Tahara et al., 2015; Sasaki et al., 2016).

This study examined whether the time lag of the circadian clock system would be improved by the presence or absence of exercise, which is relevant to the central and peripheral clocks and can be incorporated into daily life. As a model that reflects the phenomenon of SJL, our previous study reported a model in which the LD conditions recede only on 2 days that fall on holidays (Haraguchi et al., 2021). A previous paper has demonstrated that wheel-running exercise delayed both general rhythm and peripheral rhythm such as in the liver and spleen without affecting SCN rhythm (Pendergast et al., 2014). This paper also revealed that high fat diet (HFD) delayed general activity but advanced peripheral rhythm without affecting SCN rhythm (Pendergast et al., 2014). The discrepancy between activity rhythm (*in vivo* evaluation) and clock gene rhythm (*ex vivo* evaluation) may be due to methodological differences.

Therefore, in the present experiment, the effect of wheel-running exercise on SJL was evaluated by general activity and peripheral clock imaging *in vivo* (Tahara et al., 2012) under normal diet (ND) and HFD feeding. We used SJL model as a basis to evaluate how the disturbance of the body clock is affected by the presence or absence of wheel-running activity by means of behavioral rhythms, which largely reflect the influence of the SCN, the central clock, and rhythms of clock gene expression in the peripheral organs. In addition to the above experiment, we want to know whether wheel-running exercise before and/or after LD advance shift could be equally effective on synchronization.

## Materials and Methods

### Animals

Male Institute for Cancer Research (ICR) mice over 7 weeks of age and PER2: LUCIFERASE (LUC) knock-in mice (background: ICR mice) were kept in an animal chamber (temperature, 22 ± 2°C; humidity, 60 ± 5%) with an area sensor or a wheel-running device. The reason for using only male mice is that the activity level of the locomotor rhythm in female hamsters is known to change synchronously with the 4-day estrus cycle (Takahashi and Menaker, 1980). In the current study we used only male mice to investigate the relationship between the exercise and body clock without the effect of estrus cycle. The ICR mice were used in experiments 1, 3, and 6, while the PER2:LUC knock-in mice were used in experiment 2, 4, and 5. The mice were fed a normal diet (ND: EF; Oriental Yeast Company, Tokyo, Japan) in Experiments 1, 2, 5, and 6 and a high-fat diet (HFD; D12451; Research Diets Inc., NJ, United States) in Experiment 3, 4 and were given food and water *ad libitum* before and during the experiments. In all experiments, ND and HFD feeding was done approximately 2 weeks before the experiment. The heterozygous PER2:LUC knock-in mice used in this study were bred in our laboratory and raised as described previously (Tahara et al., 2012). The experimental procedures conformed to the “Basic Guidelines for the Appropriate Conduct of Animal Experiments and Related Activities in Academic Research Institutions” (Ministry of Education, Culture, Sports, Science and Technology of Japan) and were approved by the Animal Experiment Committee of the Faculty of Science and Engineering, Waseda University (2019-A078).

### Locomotor Activity and Wheel-Running Analysis

Locomotor activity was measured using an infrared sensor (area sensor) (F5B; Omron Corporation, Tokyo, Japan) for general activity rhythms, and wheel-running activity was measured using a running wheel (PS, ABS, and steel made with diameter of 120 mm and a width of 60 mm) (Silent Wheel Flat; sanco Corporation, Tokyo, Japan) for spontaneous wheel-running rhythms. Locomotor activity and wheel-running rhythms were analyzed using ClockLab software (Actimetrics, Wilmette, IL, United States) and are shown as double-plot actograms calculated by the number of sensor counts/6 min. The locomotor activity onset time was detected as the transition from the 6-h period of inactivity to the 6-h period of high activity. The free-running rhythm periods of the activity rhythms were calculated by a chi-squared test.

### Light Condition

We defined 8:00 am to 8:00 pm as the normal LD condition, and zeitgeber time 0 (ZT0) and ZT12 as the time when the lights were on and off, respectively. All mice in this study were kept in a sensor-equipped chamber, and the LD conditions were controlled for each experiment. First, in Experiments 1∼4, we delayed the LD condition by 6 h only on two consecutive days corresponding to Saturday and Sunday, defined as the SJL condition, and returned to the normal LD condition on Monday (Haraguchi et al., 2021). There are several previous studies that use LD cycle changes mimicking SJL. One study examined acclimation to a new light environment by movement, with an 8-h phase delay and phase advance over a short period of time. However, with the 8-h shift, the range of movement was so large that the activity onset time did not return to normal even after more than two weeks (Yamanaka et al., 2008). On the other hand, one study examined the estrus cycle of female mice using a regular 3-h delay in the onset of the dark period as a model and found that the 3-h shift had a small range of movement, so that the activity onset time had returned without any action on the fifth day after the light conditions were restored (Takasu et al., 2015). This was not appropriate as a model for SJL lag with a chronic body clock, because it would have already improved on Friday. Based on these papers, we used the model using the 6-h shift of LD conditions as the SJL model. In Experiment 5, the animals were reared under normal LD conditions, and then the light conditions were advanced for 6 h by changing of the start of the dark period. In Experiment 6, the animals were kept under DD condition.

### *In vivo* Monitoring Analysis

In our previous study, we developed a protocol and analytical method to monitor bioluminescence oscillations in peripheral tissues (Tahara et al., 2012). Briefly, the PER2:LUC mice were anesthetized with a mixture of isoflurane (Mylan Inc., Tokyo, Japan) and enriched oxygen and then subcutaneously injected with potassium salt of D-luciferin (15 mg/kg; Promega, Madison, WI, United States) at the base of the neck. Ten min after the injection, dorsal and ventral photographs were taken using an *in vivo* imaging system (Perkin Elmer, Waltham, MA, United States) with an exposure time of 1 min each. The bioluminescence of the mice was measured six times consecutively (at 4-h intervals) for 24 h starting at 17:00 in real time. The mice were returned to their cages after each imaging session and quickly recovered from the isoflurane anesthesia. Photon counts were analyzed using Living Image 3.2 software (Perkin Elmer). For each organ, the daily average photon/min value was converted to 100% and applied to represent the daily bioluminescence rhythm. Normalized data rhythmicity and acrophase were calculated using a single cosinor analysis software (Acro.exe version 3.5).

### Data Analysis

All values are expressed as mean ± standard error of the mean (SEM). The statistical analysis was performed using GraphPad Prism (GraphPad Software, San Diego, CA, United States). Initially, whether the data were normally or non-normally distributed was assessed by the Kolmogorov-Smirnov normality test/one-sample *t*-test and Bartlett’s test for *F*-test/comparison of variances. The parametric analysis was performed using the t-test, one-way analysis of variance (ANOVA), or repeated measures ANOVA with Tukey’s multiple comparisons test, while the non-parametric analysis was performed using the Mann-Whitney test, Kruskal-Wallis test with Dunn’s multiple comparisons test.

## Results

### Experiment 1: Effect of Wheel-Running Exercise on the Behavioral Rhythm in the Social Jet Lag Condition During Normal Diet Feeding Condition

**FIGURE 1:**
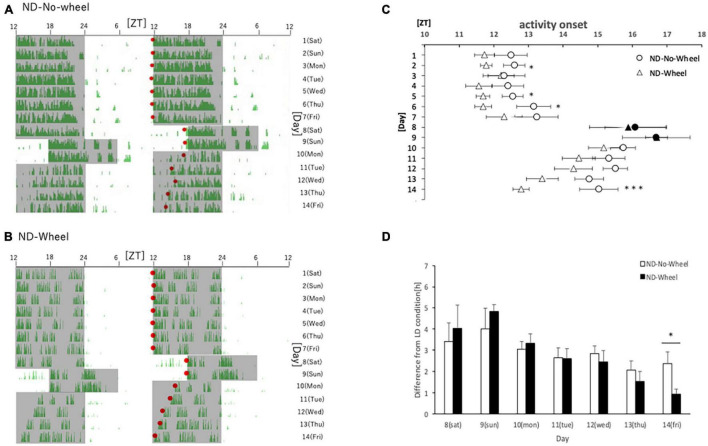
Behavioral rhythms in the SJL condition during ND feeding. **(A)** Typical double-plot actogram of behavior measured by infrared sensor during one week of rearing under the normal LD condition (days 1–7) and one week of rearing under the SJL condition (days 8–14). On days 8 and 9, which are Saturdays and Sundays, the LD conditions were set back by 6 h. **(B)** Double-plot actogram of behavioral rhythms measured with a wheel-running device on a similar schedule. **(C)** Activity onset time was calculated from the daily activity rhythms and averaged per group. Saturdays and Sundays with different start of the dark period are indicated by black marks. Data are presented as mean ± standard error of the mean (ND-No-Wheel, *n* = 5; ND-Wheel, *n* = 9). **(D)** Difference between mean activity onset time during the first half of the week (day 1–7) and activity onset time during the SJL condition (day 8–14). *p < 0.05, ***p < 0.001 (*t*-test).

First, we examined the activity rhythms of mice fed with ND in the SJL condition. We measured the amount of behavior under the rearing conditions that included a temporary delay of the LD condition for 2 days (Saturday and Sunday) as shown in a previous study (Haraguchi et al., 2021). After being kept under normal LD conditions for one week, the mice experienced a delay in light conditions for 2 days, and their recovery from the delay was observed over the next 5 days. We found that the area sensor (No-Wheel) mice without the wheel-running device were affected by the 2-days delay, and their activity onset was delayed even after returning to the normal LD condition. They did not recover to the normal activity onset, ZT12, by the fifth day (Friday) (Figures 1A,C). On the other hand, the mice with the wheel-running device showed a similar delay in the active period during the five weekdays but recovered to the same level of activity onset time as in the normal state on the fifth day (Figures 1B,D). We found that the activity onset time on the fifth day after experiencing the SJL condition was significantly earlier in the Wheel group (Figure 1C). However, the wheel-running measurement group tended to start its activity earlier than the area sensor measurement group, even during normal rearing (Figure 1C), we calculated and compared the delay in the activity onset time in the second half of the day relative to the average activity onset time in the first 7 days (Figure 1D). In both groups, the delay to the LD condition was large on Saturdays and Sundays when the light condition was delayed in the SJL condition, and it recovered over time on the following five weekdays. On all weekdays, the delay time for the first 7 days was smaller for the Wheel group, and it was significantly smaller on Friday. These results indicate that exercise by the wheel-running mice stopped the delay in the active phase caused by the SJL condition.

### Experiment 2: Effect of Wheel-Running Exercise on *Per2* Gene Expression Rhythm in Peripheral Organs in the Social Jet Lag Condition

**FIGURE 2:**
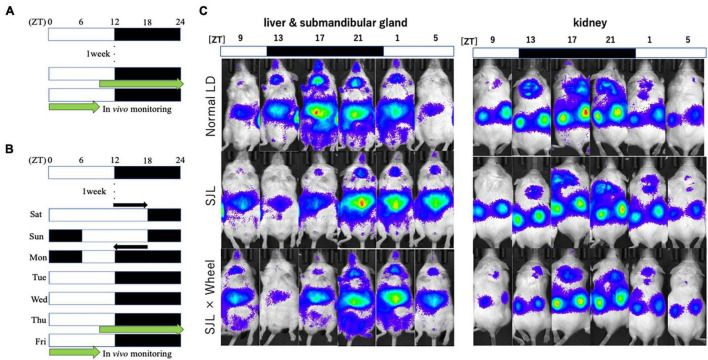
Experimental protocol and representative images of the *in vivo* imaging system. **(A)** Protocol of normal LD condition. **(B)** Protocol of SJL condition. **(C)** Representative images of *in vivo* PER2:LUCIFERASE (PER2:LUC) bioluminescence showing PER2:LUC expression rhythm as luminescence in the kidney, liver, and submandibular gland.

**FIGURE 3:**
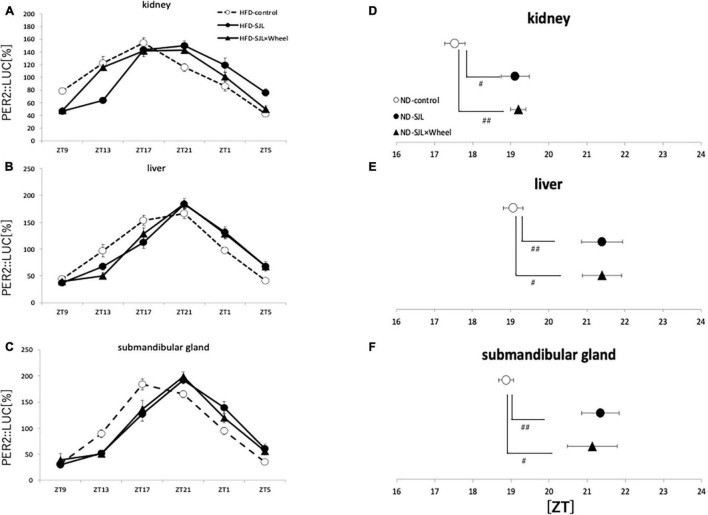
Wave form and acrophase of PER2:LUC expression rhythm in each organ in LD or SJL conditions during ND feeding. **(A–C)** Waveforms for each group in kidney, liver, and submandibular gland. The group raised under normal LD conditions was designated as the control. **(D–F)** Acrophase for each group in kidney, liver, and submandibular gland. Data are presented as mean ± standard error of the mean (ND-control, *n* = 6; ND-SJL, *n* = 7; ND-SJL × Wheel, *n* = 6). #*p* < 0.05, ##*p* < 0.01 (Kruskal-Wallis test with Dunn’s multiple comparisons test).

From Experiment 1, we found that the presence of wheel-running altered the activity rhythms linked to changes in light conditions in mice. Therefore, in Experiment 2, we measured the expression rhythm of PER2:LUC in peripheral organs on Thursday, the fourth day after experiencing SJL under the same light conditions as in Experiment 1. Measurements were performed using an *in vivo* imaging system as reported in previous studies, and PER2:LUC levels in the kidney, liver, and submandibular gland were measured as luminescence at six points over 24 h (Figures 2A–C), similar to previous paper (Tahara et al., 2012). The results showed that the expression rhythm of the PER2:LUC on Thursday was significantly delayed in the SJL group compared to the LD group that did not experience SJL. In addition, the same measurement was performed for SJL × Wheel and it was found that the phase was delayed to the same extent as in the SJL group (Figures 3A–F). Therefore, it was found that the exercise of mice with the wheel-running device did not affect the expression of clock genes in peripheral organs under SJL conditions.

### Experiment 3: Behavioral Rhythm of Social Jet Lag Model Mice During HFD Feeding Condition

**FIGURE 4:**
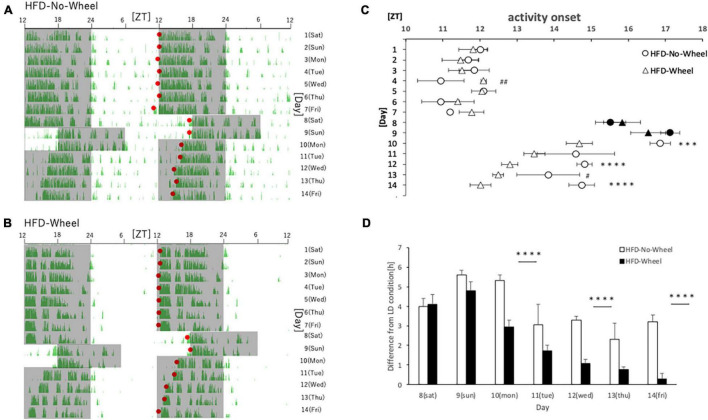
Behavioral rhythm in the SJL condition during HFD feeding. **(A)** Typical double-plot actogram of behavior measured by the infrared sensor during one week of rearing under the normal LD condition (days 1–7) and one week of rearing under the SJL condition (days 8–14). On days 8 and 9, which are Saturdays and Sundays, the LD conditions were set back by 6 h. **(B)** Double-plot actogram of behavioral rhythms measured with a wheel-running device on a similar schedule. **(C)** Activity onset time was calculated from the daily activity rhythms and averaged per group. Saturdays and Sundays with different start of the dark period are indicated by black marks. Data are presented as mean ± standard error of the mean (HFD-No-Wheel, *n* = 10; HFD-Wheel, *n* = 8). **(D)** Difference between mean activity onset time during the first week (days 1–7) and activity onset time during the SJL condition (day 8–14). ***p < 0.001, ****p < 0.0001 (*t*-test); #*p* < 0.05, ##*p* < 0.01 (Mann-Whitney test).

In Experiment 3 and 4, mice were fed with HFD. This is because the HFD itself is known to disrupt the behavioral and molecular circadian rhythms of mice, and we thought that combining this with the SJL condition would allow us to reproduce a more severe SJL condition (Kohsaka et al., 2007). In this experiment, the protocol was the same as that in Experiment 1, but the food was changed to an HFD for verification. As in Experiment 1, we found a difference in the recovery of the starting time of activities between the groups with and without the wheel on five weekdays (Figures 4A,B), and when the values were compared between the groups, a significant difference was found on most weekdays (Figure 4C). In addition, as in Experiment 1, when the animals were reared under normal LD conditions, there was a tendency for the Wheel group to start their activities earlier overall. Therefore, when we evaluated the onset time of the activity by the difference from the average onset time of the activity during the first seven days, we found a significant difference in the onset time of the activity on Monday, Wednesday, and Friday (Figure 4D). In the present experiment, wheel-running suppressed weight gain during HFD feeding which increases food intake itself (Supplementary Figure 1). Thus, the effect of wheel-running exercise is well observed. Therefore, effect of wheel-running exercise in well approved. Overall, there were more days in which the activity rhythm was significantly changed by the presence or absence of wheel-running in Experiment 3 than in Experiment 1. This result indicates that the recovery effect of the wheel-running exercise on the delay of the body clock caused by SJL was more pronounced under the HFD condition.

### Experiment 4: *Per2* Gene Expression Rhythm of Social Jet Lag Model Mice During HFD Feeding Condition

**FIGURE 5:**
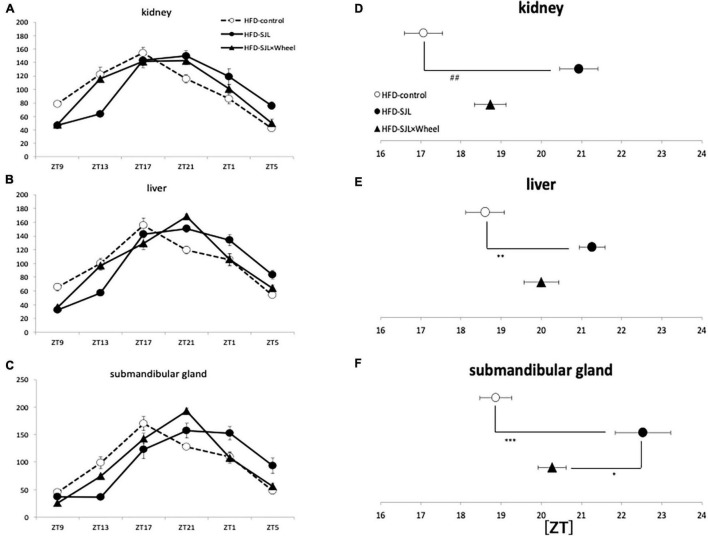
Wave form and acrophase of PER2:LUC expression rhythm in each organ in LD or SJL conditions during HFD feeding. **(A–C)** Waveforms for each group in kidney, liver, and submandibular gland. The group raised under normal LD conditions was designated as the control. **(D–F)** Acrophase for each group in kidney, liver, and submandibular gland. Data are presented as mean ± standard error of the mean (ND-control, *n* = 6; ND-SJL, *n* = 6; ND-SJL × Wheel, *n* = 6). *p < 0.05, **p < 0.01, ***p < 0.001 (one-way analysis of variance (ANOVA) with Tukey’s multiple comparisons test); ##*p* < 0.01 (Kruskal-Wallis test with Dunn’s multiple comparisons test).

As in Experiment 3, the expression rhythm of PER2:LUC in peripheral organs during HFD feeding was examined and compared with the results of Experiment 2. As in Experiment 2, the expression rhythm of the PER2:LUC was significantly delayed in the SJL group compared to the LD group. However, unlike the results of Experiment 2, when the SJL × Wheel group was compared with the LD group, there was no significant delay in any of the organs (kidney, liver, and submandibular gland), indicating an improvement in the phase delay compared with the SJL without wheel group (Figures 5A–F). Especially in the submandibular gland, the improvement effect was more pronounced, because the phase was located significantly earlier than that in the SJL without wheel group (Figures 5C,F). We observed changes in the PER2:LUC rhythm in the peripheral organs under SJL conditions by wheel running, which did not appear in the ND. This indicates that HFD feeding causes greater disturbance in the expression of clock genes in peripheral organs as well as in behavior under SJL conditions, and that wheel-running exercise is more effective under these conditions.

### Experiment 5: Changes in *Per2* Gene Expression in the Peripheral Organs in Response to 6 h of Advance Light Condition

**FIGURE 6:**
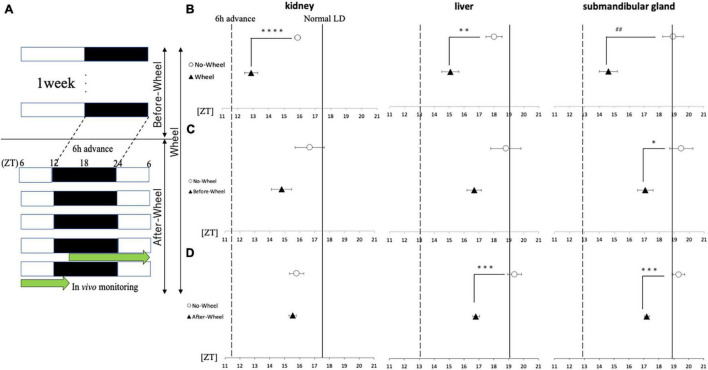
Acrophase of PER2:LUC expression rhythm in each organ in response to 6 h of advance light conditions. **(A)** Protocols of three trials with different light conditions and wheel-running timings. **(B–D)** Comparison of the peak time of PER2:LUC expression (acrophase) in each trial. The solid line shows the acrophase under normal LD conditions with no wheel-running, and the wavy line shows the value advanced from that point by 6 h, or the acrophase that should be aimed for. Data are presented as mean ± standard error of the mean (*n* = 6 for each group for all trials). *p < 0.05, **p < 0.01, ***p < 0.001, ****p < 0.0001 (t-test); ##*p* < 0.01 (Mann-Whitney test).

Experiments 2 and 4 showed that wheel-running exercise under SJL light conditions was effective in both behavioral rhythms and clock gene expression in peripheral organs when the animals were fed with HFD, whereas no effect was observed in peripheral organs of mice fed with ND. This may be due to the small effect of SJL in the ND and the fact that the SJL model is a complex model with a series of delays and advances. Therefore, in order to evaluate the effect of wheel-running exercise on the body clock in more detail, the effect of wheel-running exercise was examined using a simple model in which the light condition was advanced for 6 h instead of the SJL model (Figure 6A). When comparing the phase of the peripheral clock of mice with/without wheel-running exercise, the kidney (ZT18.8 ± 0.1 for Wheel, ZT17.5 ± 0.3 for No-Wheel), liver (ZT19.2 ± 0.4 for Wheel, ZT19.0 ± 0.3 for No-Wheel) and submandibular gland (ZT19.5 ± 0.3 for Wheel, ZT18.9 ± 0.2 for No-Wheel) clock phases were observed. Thus, wheel-running caused a small delay of peripheral clock. The results of the 6-h advanced light condition showed that phase of PER2:LUC rhythm in all three organs was significantly advanced in the Wheel compared to the No-Wheel group (Figure 6B).

Next, using a 6-h forward protocol, we prepared a group with a wheel only before the time shift (Before-Wheel) and a group with a wheel only after the time shift (After-Wheel) and compared them with the No-Wheel group to examine differences in the timing of the wheel movements (Figures 6C,D). The results of the experiment in which the wheel-running was restricted to only before or after the time shift showed different effects on different organs. In the kidney, there was no significant difference in the Before-Wheel and After-Wheel groups versus the No-Wheel group (Figures 6C,D). In the liver, there was no difference between the Before-Wheel group and the No-Wheel group, but there was a significant difference between the After-Wheel group and the No-Wheel group (Figures 6C,D). In the submandibular gland, there was a significant difference in treatment compared to the No-Wheel group in both trials (Figures 6C,D). These results indicate that wheel-running helped the body internal clock to synchronize to changes in the advance direction; in particular, wheel-running immediately after the LD shift may be more important than wheel-running before the LD shift.

### Experiment 6: Free-Run Period Under the DD Condition

Experiment 5 demonstrated that wheel-running alters the expression rhythm of under changing light conditions. Therefore, here we examined whether the wheel-running exercise affected the internal clock of the mouse itself regardless of the change in light. By rearing mice in the DD condition and measuring their behavior, we investigated how the circadian free-running rhythm changed depending on wheel-running status.

**FIGURE 7:**
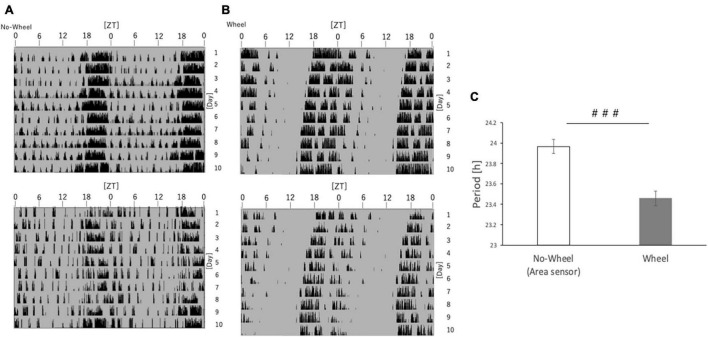
Free-running cycle of mice under DD condition. **(A)** Representative double-plot actograms of the behavior of mice reared under DD conditions for 10 days as measured by an infrared sensor. **(B)** Representative double-plot actogram of behavior measured by a wheel-running device during 10 days of rearing under DD condition. **(C)** Free-run cycle calculated from behavioral rhythms. Data are presented as mean ± standard error of the mean (No-Wheel, *n* = 9; Wheel, *n* = 7). ###*p* < 0.001 (Mann-Whitney test).

As a result, mice in the measurement system without the running-wheel showed a free-running period of approximately 24.00, while those in the group with the wheel showed a free-running period of nearly 23.50 (Figure 7). In other words, the free-running cycle in the absence of light stimulation was significantly different depending on the presence or absence of wheel-running. This result shows that one of the mechanisms by which the presence of wheel-running helps synchronize in the advance direction, as in Experiments 1 and 5, is that it is possible the running-wheel exercise itself shortens the cycle of the circadian rhythm regardless of the light condition, thereby facilitating adaptation to the phase advance.

## Discussion

In this study, we examined the effect of the wheel-running exercise on circadian clock delay using SJL model mice. In Experiments 1 and 3, we measured behavioral rhythms after SJL condition and found that the recovery of delayed behavioral rhythms was faster in the wheel-running group than in the area sensor (No-Wheel) group. In addition, Experiment 2 and 4 examined the rhythms of PER2:LUC expression in the peripheral organs under SJL conditions and found that the delayed amelioration effect of wheel-running exercise was observed only during HFD feeding. Experiments 5 and 6, which did not use the SJL model, showed that wheel-running promoted the synchronization of the rhythm of PER2:LUC expression in the peripheral organs to the advance shift and that wheel-running shortened the behavioral cycle of mice under DD conditions.

One of the factors that may influence the adaptation of wheel-running to the shift of the body clock, such as SJL and 6-h forward shift, is the change in the free-running cycle due to the presence of wheel-running, as shown in Experiment 6. In Experiment 6, the free-running period in the DD condition was significantly shorter in the presence of wheel-running than in the No-Wheel group. This may be due to the increase in locomotion of the mice in the presence of wheel-running. Previous studies reported that wheel-running caused a shorter free-running period in rats (Shioiri et al., 1991). and mice (Edgar et al., 1991), and the present results are consistent with these findings. On the other hand, in a study using the 129S6 back-crossed with C57BL/6 strain, it was reported that the free-running period was rather shortened by removing the running wheel from the condition in which the running wheel was attached for 175 days (Harrington et al., 2007), so the effect of running wheel exercise on shortening the free-running period may depend on mouse strain and wheel-running period. Therefore, it is highly likely that the wheel-running exercise itself affects the body clock. A possible mechanism for this is the influence of serotonin. In fact, a previous study described the importance of serotonin in shortening cycles (Shioiri et al., 1991).

We showed that the free-running period length was significantly shorter in mice with a running wheel than without a wheel, thus pointing to the possibility that this shortness causes the faster adjustment to the advanced LD cycle. This hypothesis in turn raises a possibility that mice with a running wheel adjust more slowly when LD cycle is delayed. If the phase of the mice with a running wheel was only slightly delayed compared to that without a wheel on day 9 (Sunday), the advanced activity onset in mice with a running wheel on day 14 (Friday) was just caused by the advanced phase of its internal clock on day 9, not a faster adjustment by the wheel-running exercise. When we checked the phase of activity rhythm on day 9, no significant phase differences were observed between Wheel groups and No-Wheel groups (Figures 1, 4), suggesting the preference of the shorter free-running mechanism rather than the small phase-delay mechanism. To clarify this argument, we need to check the phase of the peripheral clock on day 9 (Sunday)in SJL model mice in the future.

Previous studies reported that serotonin is involved in the pathway of arousal-induced entrainment in the SCN, and the injection of 8-OH-DPAT, an agonist of serotonin 1A/7 receptors, into hamsters caused a phase change in their behavioral rhythm (Cutrera et al., 1994; Horikawa et al., 2000). It was also reported that the expressions of *Per1* and *Per2* in the SCN were similarly decreased, which is the same as that obtained by the stimulation of wheel-running (Cuesta et al., 2008). In relation to wheel-running exercise, it has been reported that serotonergic afferent may be part of an activity-dependent entrainment mechanism since spontaneous exercise in rodents increases the serotonin content of the SCN and serotonergic agonists alter the phase of the circadian clock in a manner very similar to spontaneous intense exercise (Edgar et al., 1997). Therefore, in future experiment, we should examine the effect of blockade of serotonergic system on wheel-running exercise induced recovery of SJL.

It is also possible that the light sensitivity was altered by the wheel-running. However, in Experiments 1 and 3, a 6-h delay in light conditions on Saturday and Sunday delayed the start of activity for 3.5 to 4 h on Saturday and 4 to 5.5 h on Sunday, whereas there was no significant difference in the degree of delay between the No-Wheel group and the Wheel group. Therefore, the sensitivity to light may not be changed by wheel-running.

Stress is another factor that is often discussed in conjunction with exercise. Previous studies reported that forced exercise during inactive periods synchronized the peripheral clock by increasing corticosterone and noradrenaline levels (Sasaki et al., 2016). Several studies reported that the pathways involved in exercise-induced peripheral clock entrainment are related to both exercise and stress (Chennaoui et al., 2002; Fediuc et al., 2006; Stranahan et al., 2008). However, in the present experiment, we only set up the wheel-running machine and did not force the subjects to exercise, so the stress was low. Previous studies reported that the increase in corticosterone concentration due to exercise depends on exercise type and intensity and that these effects were slightly observed in spontaneous wheel-running (Fediuc et al., 2006; Tahara et al., 2017a), so the stress is not considered to be high enough to explain wheel-running induced facilitation of the body clock synchronization.

In addition, it is possible that this is due to an increase in body temperature caused by exercise. Although the circadian clock system is quite resistant to changes in ambient temperature, sustained or transient temperature changes synchronize the expression of PER2:LUC in the peripheral tissues (Isojima et al., 2009; Narasimamurthy and Virshup, 2017). Under high-temperature night conditions (24°C daytime, 37°C nighttime) for 6 days, the phase of the clock gene expression rhythm changes in the liver of mice (Brown et al., 2002). It is conceivable that wheel-running may have caused a continuous change in body temperature in the present experiment; our unpublished data show that the wheel-running exercise increased the average body temperature of mice from 37.5 to 38.5°C, but the room temperature was low, about 22°C and the change of body temperature itself was small, so it is unlikely to be a useful factor in promoting a phase advance.

Comparing Experiments 1and 2 with ND, the effects of the wheel-running exercise were more pronounced in Experiments 3 and 4 with HFD. This may be due to the fact that the HFD increases the disruption of the body clock caused by the SJL model. One possible mechanism of this phenomenon may increase in oxidative stress (Kohsaka et al., 2007). Physiological stresses, such as oxidative stress, can cause synchronization of the circadian clock, and the addition of sudden light changes to this stress may have caused a greater disruption of the body clock (Tahara et al., 2017a). It is possible that the ameliorating effect of wheel-running on the body clock, which had been greatly disrupted because of oxidative stress and other overall deterioration of metabolism in mice, advanced the active phase.

In the current experiments, we assessed the effect of wheel-running exercise on peripheral clock and behavioral rhythms under ND or HFD. The delay value of locomotor activity rhythm by 6-hr LD delay shift was similar between ND and HFD condition (Figures 1, 4). On the other hand, peripheral clocks especially in kidney and submandibular gland showed more delayed phase in HFD conditions compared with ND (Figures 3, 5). In this experiment, wheel-running exercise clearly improved the SJL under HFD condition but not under ND condition. Thus, such delay of peripheral clock by HFD feeding may be further good model of SJL.

In the simple advance model, we found wheel-running exercise clearly potentiated phase advance of peripheral clocks. L. Leise examined phase advance of behavioral rhythm and peripheral clock rhythm in aged mice by LD advance and found that wheel-running facilitated the synchronization of behavior rhythm and peripheral clock gene rhythm (Leise et al., 2013). The present result with this paper strongly suggests the facilitating effect of wheel-running on synchronization in LD advance model. There is a misleading study which evaluated the effect of wheel-running exercise on behavior rhythm *in vivo* and peripheral organ rhythm *ex vivo* under HFD feeding (Pendergast et al., 2014). This research evaluates peripheral clocks in organs *ex vivo*, which may cause discrepancies with *in vivo* results such as behavioral rhythms, and the results may differ between organs. HFD caused phase delay in spleen clocks and locomotor activity rhythm, but phase advance in liver clocks (Pendergast et al., 2014). Therefore, in this study, we focused on eliminating this discrepancy by conducting all assessments *in vivo*. As a result, the expression rhythms of the PER2:LUC in all organs were consistent with the results of the behavioral rhythms. Thus, in the current experiment, we did not use the *ex vivo* approach, however in the future experiment, we should evaluate the effect of wheel-running exercise on SCN clocks in *ex vivo*.

In the present study, we examined the difference in synchronization effects of wheel-running timing using a 6-h forward shift, which is simpler than SJL. In both the Before-Wheel and After-Wheel trials, there was a tendency to move forward compared to the No-Wheel group, but the range of forward movement was smaller than in the Wheel trial where the wheel was always on. This suggests that the length of time spent running with the wheel is important for phase advancement, which is consistent with previous studies. In the submandibular gland, there was a significant advance in both Before-Wheel and After-Wheel trials. Previous studies have reported that the sympathetic nervous system is associated with the expression of clock genes in the submandibular gland (Tahara et al., 2017b). Therefore, the present results indicate that the sympathetic nervous system of mice may be altered by the wheel-running exercise, a change that can be seen in the effect of wheel-running the wheel before shift, and that increased sympathetic activity may be maintained for several days after LD shift.

In conclusion, our findings suggest that wheel-running exercise shortens the activity cycle and helps the circadian system synchronize with the advance direction change. In addition, the effective use of forward directional synchronization by the wheel-running exercise may have improved the disturbance of the body clock caused by SJL and shift work. By applying this method to modern society, it may be possible to improve the time lag between the activity time required by society and the activity time of the intrinsic body clock through exercise and maintain the body clock by quickly adapting to the changed time schedule. In addition, since the pathway of the circadian clock system during exercise involves serotonin, and the phase is likely to be advanced by arousal (Smith, 2002; Cuesta et al., 2008), serotonergic drugs and caffeine, which has arousing effects, may also help to eliminate SJL.

## Data Availability Statement

The original contributions presented in the study are included in the article/Supplementary Material, further inquiries can be directed to the corresponding author.

## Ethics Statement

The animal study was reviewed and approved by Approved by the Animal Experiment Committee of the Faculty of Science and Engineering, Waseda University (2019-A078).

## Author Contributions

SO performed the experiments, analyzed data, and wrote the manuscript. SC and HS performed the experiments. AH performed the experiments and interpreted data. SS planned experiments, interpreted data and made manuscript revisions. All authors contributed to the article and approved the submitted version.

## Conflict of Interest

The authors declare that the research was conducted in the absence of any commercial or financial relationships that could be construed as a potential conflict of interest.

## Publisher’s Note

All claims expressed in this article are solely those of the authors and do not necessarily represent those of their affiliated organizations, or those of the publisher, the editors and the reviewers. Any product that may be evaluated in this article, or claim that may be made by its manufacturer, is not guaranteed or endorsed by the publisher.
