# Supplementary material for: Wheel-Running Facilitates Phase Advances in Locomotor and Peripheral Circadian Rhythm in Social Jet Lag Model Mice
Source: Front Physiol. 2022 Feb 16;13:821199. doi: 10.3389/fphys.2022.821199 (PMC8890682; doi:10.3389/fphys.2022.821199)
Supplement: Supplementary file 1 [file Image_1.pdf]

Supplementary Material

1. Body weight and food consumption during high fat diet (HFD) feeding

In order to clarify the wheel running effect on body, weight gain and food consumption volume were compared between Wheel and No-Wheel groups. A two-way analysis of variance (ANOVA) with repeated testing was performed after ensuring that all data satisfied normality. The results showed that there was no significant difference in body weight for all four weeks. In terms of feeding volume, it was found that the Wheel group fed significantly more than the No-Wheel group at week4 (Sidak test). These results suggest that present protocol of wheel running is enough to show physiological effect.

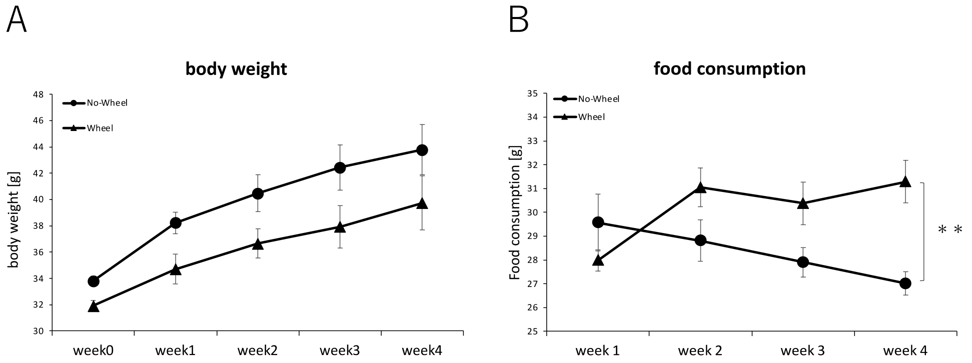

Supplementary Figure 1. Weekly weight change (A) and food consumption volume (B) during HFD feeding. Data are presented as mean ± standard error of the mean (No-Wheel, n = 9; Wheel, n = 7), $^{**}p<0.01$ (two-way analysis of variance (ANOVA) with Sidak multiple comparisons test)
